# Pitfalls of the AdaptivCRT algorithm for effective pacing: Optimization using the EffectivCRT algorithm data

**DOI:** 10.1016/j.hrcr.2024.02.004

**Published:** 2024-02-10

**Authors:** Nobuhiko Ueda, Satoshi Oka, Kohei Ishibashi, Takeshi Kitai, Chisato Izumi, Kengo Kusano

**Affiliations:** Department of Cardiovascular Medicine, National Cerebral and Cardiovascular Center, Suita, Japan

**Keywords:** Cardiac resynchronization therapy, EffectivCRT, Left ventricular pacing, Conduction delay, Latency

**Key Teaching Points**

• The adaptivCRT (aCRT) algorithm is effective for patients undergoing cardiac resynchronization therapy with normal atrioventricular (AV) conduction, and a higher percentage of synchronized left ventricular (LV) pacing is associated with favorable outcomes.
• Even though LV pacing was delivered earlier than the intrinsic rhythm with aCRT, pacing could be ineffective owing to paced conduction delay at the pacing site.
• Manual AV optimization, instead of aCRT based on data from the effectivCRT algorithm, could be an alternative method for effective pacing.

## Introduction

The adaptivCRT (aCRT) algorithm was developed to provide continuous optimization and synchronized left ventricular (LV) pacing to right ventricular (RV) sensing.^1^, ^2^, ^3^ It is effective in patients with normal atrioventricular (AV) conduction, and a higher percentage of synchronized LV (sLV) pacing is associated with superior clinical outcomes.^4^^,^^5^

The effectivCRT (eCRT) algorithm can quantify LV pacing using a beat-to-beat analysis of the paced morphology of an electrogram (EGM) to assess suboptimal LV pacing. It can accurately classify effective pacing beats, pseudofusion beats, and beats with a loss of LV capture.^6^ On the other hand, though patients with intraventricular conduction disturbance could find it hard to respond to cardiac resynchronization therapy (CRT), QRS axis shift from left axis deviation to right axis deviation on surface electrocardiogram (ECG) was a predictor of response to CRT.^7^ In addition, LV-paced conduction disturbances are associated with unfavorable outcomes.^8^

This report presents a pitfall of the aCRT algorithm in patients with a high percentage of synchronized LV pacing and an alternative method for effective pacing based on the data produced by the eCRT algorithm.

## Case report

**Figure 1:**
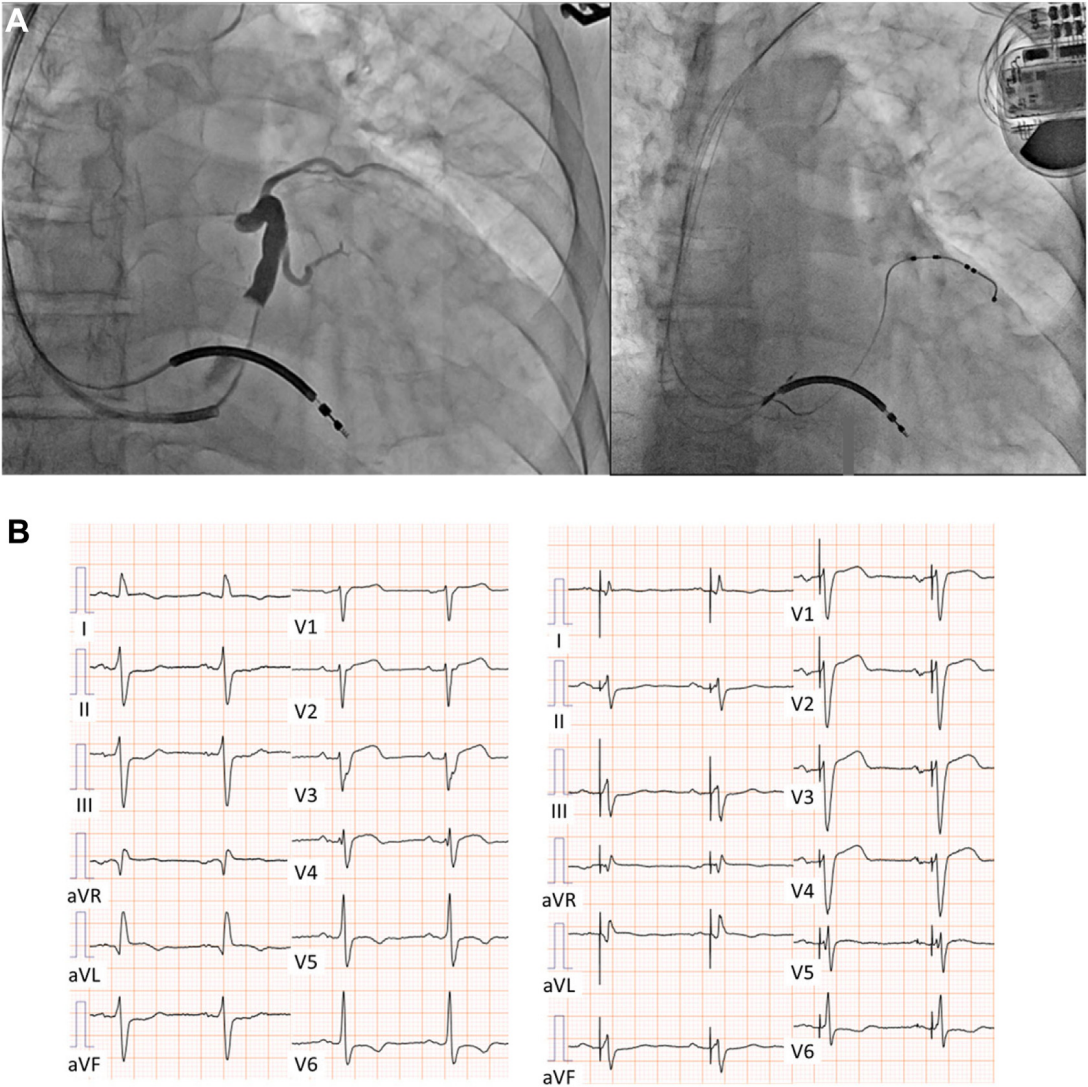
Chest radiography and electrocardiogram (ECG). **A:** Coronary sinus angiogram and fluoroscopic radiography image after cardiac resynchronization therapy implantation. Left ventricular, right ventricular (RV), and right atrial lead positioning at the anterolateral coronary sinus branch, RV apex, and right atrial septum, respectively. **B:** ECG showing a sinus rhythm, left axis deviation, intraventricular conduction disturbance, and a QRS duration of 145 ms.

A 72-year-old male patient with ischemic cardiomyopathy was referred to our hospital with dyspnea. A percutaneous coronary intervention was performed for the left anterior descending coronary artery 3 times and for the right coronary artery once. The patient had a history of hospitalization owing to heart failure. Transthoracic echocardiography revealed an LV ejection fraction of 30%. An ECG revealed sinus rhythm, left axis deviation, intraventricular conduction disturbance, and a QRS duration of 145 ms. Single-photon emission computed tomography showed perfusion defects in the anterior and apex regions. The patient exhibited New York Heart Association class III symptoms, and nonsustained ventricular tachycardia was documented. A CRT device with defibrillator (CRT-D) (Cobalt XT HF Quad; Medtronic Inc., Minneapolis, MN) was implanted at the LV, RV, and right atrial (RA) lead positions in the anterolateral coronary sinus branch, RV apex, and RA septum, respectively (Figure 1). Because the ECG of this patient showed left axis deviation, and a phrenic nerve was stimulated at the posterolateral branch, we selected the anterolateral location instead of posterolateral targets. A quadripolar lead was used, and the Q-LV intervals of LV-1, LV-2, LV-3, and LV-4 were 91, 95, 103, and 87 ms, respectively. Given the pacing thresholds of LV-2, LV-3, and LV-4 were high, LV-1 was selected as the pacing site.

**Figure 2:**
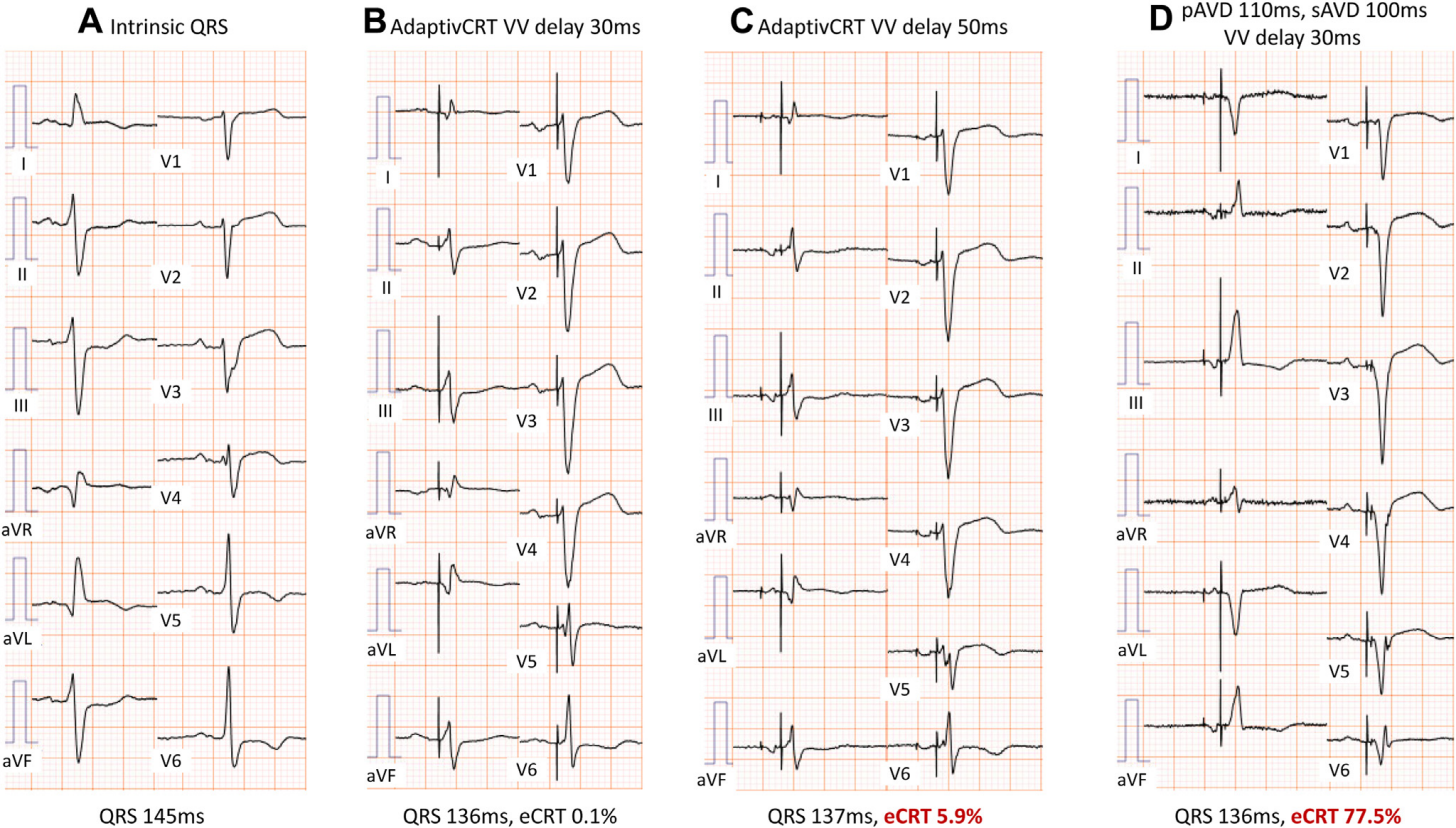
Electrocardiogram (ECG) before and after cardiac resynchronization therapy (CRT) implantation and optimization. **A:** Before CRT implantation (intrinsic QRS). **B:** Activation of adaptivCRT (aCRT). Although the QRS duration narrowed from 145 to 136 ms, the percentage of the effective CRT pacing (%e-CRT) was 0.1%. **C:** The VV delay was changed from 30 to 50 ms. There was no change in QRS duration, and %e-CRT remained low (5.9%). **D:** Deactivation of aCRT, followed by manual electrical optimization using device setting data, was performed. We programmed paced AV delay (pAVD) to 110 ms; sensed AV delay (sAVD) to 100 ms, which was 30 ms earlier than the setting by aCRT; and VV delay to 30 ms. After optimization, the ECG showed a QRS axis shift from left axis deviation to right axis deviation and Q wave in the V_5_ and V_6_ leads, and the %e-CRT improved from 5.9% to 77.5%.

After CRT-D device implantation, aCRT was initiated, as AV conduction was intact. Although the QRS duration decreased from 145 to 136 ms, the percentage of the effective CRT pacing (%e-CRT) was 0.1% (Figure 2A and 2B). As the record from the device memory showed “delayed LV activation,” we suspected that the cause of the low %e-CRT was “latency,” which indicates conduction delay from LV pacing to myocardial excitation. Other pacing polarities could not be selected because of a high pacing threshold. Next, the VV delay was changed from 30 to 50 ms 8 months after aCRT activation; however, there was no change in the QRS duration, and the %e-CRT was still low (5.9%) (Figure 2C). At this point, the percentages of biventricular and LV pacing were 1.6% and 98.3%, respectively. RV pacing did not occur and thus could not be changed by LV pacing delivery, even if the VV delay was prolonged. As effective pacing could not be delivered with aCRT, it was deactivated, and manual electrical optimization with the device setting data was performed. The device setting data showed that paced AV delay (pAVD) and sensed AVD (sAVD) were 140 and 130 ms, respectively. We programmed pAVD to 110 ms; sAVD to 100 ms, which was 30 ms earlier than the setting by aCRT 4 months after the timing of VV delay change; and VV delay to 30 ms (Figure 2D). After optimization, the ECG showed a QRS axis shift from left axis deviation to right axis deviation and Q wave in the V_5_ and V_6_ leads, %e-CRT improved from 5.9% to 77.5%, and dyspnea improved.

## Discussion

The aCRT algorithm is effective for patients undergoing CRT with normal AV conduction.^4^ A previous report showed that aCRT or prolongation of the VV delay could improve the %e-CRT.^9^^,^^10^ This report shows that these 2 methods failed to improve %e-CRT, and manual optimization was suitable for effective pacing in patients with latency at the pacing site and abundant LV pacing.

**Figure 3:**
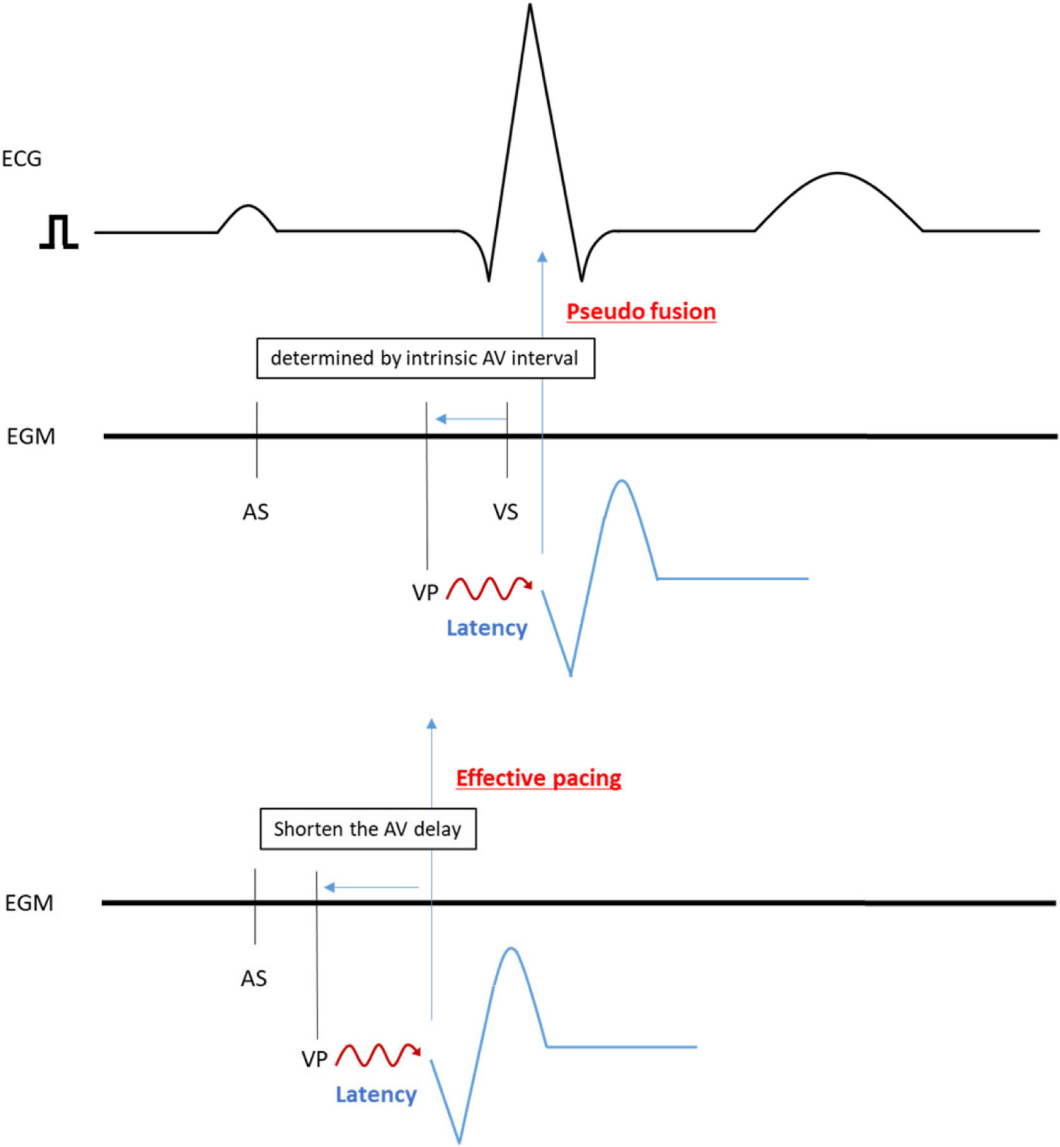
Schema of manual optimization. The adaptivCRT (aCRT) algorithm provides synchronized left ventricular (sLV) pacing to right ventricular (RV) sensing, with the atrioventricular (AV) delay calculated from the intrinsic atrial-RV sense interval (*horizontal blue arrow*). In patients with delayed left ventricular activation (latency) at the pacing site (*wavy line*), sLV pacing resulted in pseudofusion (*vertical blue arrow*; upper panel). After the deactivation of aCRT, manual electrical optimization was performed to shorten the AV delay (*horizontal blue line*; lower panel). Effective pacing was observed (*vertical blue line*).

The OLE CRT study showed that the causes of ineffective CRT pacing are latency, atrial fibrillation, and loss of capture.^11^ Changing VV delay and aCRT can effectively improve %e-CRT.^9^^,^^10^ In this case, the cause of the low %e-CRT was latency, which was recognized as “delayed LV activation” in the device data, though there were insufficient data of paced conduction delay during the CRT-D implantation. Both aCRT and changing the VV delay failed to improve the %e-CRT. At this point, almost all ventricular pacing was LV, which is a drawback of aCRT in patients with latency at the pacing site and abundant LV pacing. Pacing timing was determined only by intrinsic AV duration (in short, the algorithm provides LV pacing with AV delay calculated from the intrinsic A-RVs interval, to pace at either 70% of the measured AV interval or 40 ms before intrinsic A-RVs interval, whichever value is smaller), with or without latency as described earlier; this timing cannot be changed by changing VV delay because RV pacing is not delivered. We selected the RA lead position as RA low septum to shorten A-RVs and obtain a higher burden of LV pacing. Even a longer AV interval with the RA lead position as RAA instead of RA low septum could also cause delayed LV activation owing to latency. One solution was using manual optimization instead of aCRT (Figure 3).

There is no established method to determine the optimal setting in patients with latency at the pacing site and abundant LV pacing. The latency diagnosis and lack of an alternative pacing vector choice allowed us to select manual AV optimization using the morphology of an EGM instead of ECG morphologies, and latency appears a suitable reference for AV delay, which means shortening AV delay changed the EGM morphology. First, we programmed pAVD and sAVD 30 ms earlier than the setting by aCRT, and the EGM morphology changed to an initially negative deflection. It is unclear whether a shorter AVD would be better; however, the fusion of LV pacing and intrinsic rhythm could be better. As the GREATER-EARTH trial revealed that LV pacing (without fusion with the intrinsic rhythm) is not superior to biventricular pacing, an extremely short AVD is not suitable for cardiac synchronization.^12^ Trucco and colleagues^13^ showed that a fusion-optimized interval achieved favorable LV remodeling compared with a normal setting. The longest AVD possible for effective pacing timing could be better because fusion with an intrinsic rhythm could be ideal.^14^ In addition, the ECG showed that the QRS axis shifted from left axis deviation to right axis deviation on surface ECG; this could represent a favorable response to CRT-D in this case. As the high percentage of effective CRT pacing (%e-CRT) is associated with the CRT response, increasing %e-CRT could improve the response to CRT.^15^^,^^16^

## Conclusion

A high percentage of %e-CRT is important for effective pacing in patients undergoing CRT. Manual AV optimization based on data from the eCRT algorithm instead of aCRT could be an alternative method for effective pacing in patients with abundant LV pacing using aCRT and delayed LV activation at the pacing site.

## Disclosures

Dr Ueda has received honoraria from Medtronic Japan Co, Ltd, for providing lectures. Dr Ishibashi has received honoraria from BIOTRONIK Japan and Medtronic Japan Co, Ltd, for providing lectures. Dr Kusano received honoraria from BIOTRONIK Japan and Medtronic Japan Co, Ltd, and research grants from Medtronic Japan Co, Ltd. However, none of these were directly associated with this study. The remaining authors have no conflicts of interest to declare.

## References

[1] Krum H., Lemke B., Birnie D. (2012). A novel algorithm for individualized cardiac resynchronization therapy: rationale and design of the adaptive cardiac resynchronization therapy trial. Am Heart J.

[2] Khaykin Y., Exner D., Birnie D., Sapp J., Aggarwal S., Sambelashvili A. (2011). Adjusting the timing of left-ventricular pacing using electrocardiogram and device electrograms. EP Europace.

[3] van Gelder B.M., Bracke F.A., Meijer A., Pijls N.H. (2005). The hemodynamic effect of intrinsic conduction during left ventricular pacing as compared to biventricular pacing. J Am Coll Cardiol.

[4] Birnie D., Lemke B., Aonuma K. (2013). Clinical outcomes with synchronized left ventricular pacing: analysis of the adaptive CRT trial. Heart Rhythm.

[5] Wilkoff B.L., Filippatos G., Leclercq C. (2023). Adaptive versus conventional cardiac resynchronisation therapy in patients with heart failure (AdaptResponse): a global, prospective, randomised controlled trial. Lancet.

[6] Ghosh S., Stadler R.W., Mittal S. (2015). Automated detection of effective left-ventricular pacing: going beyond percentage pacing counters. EP Europace.

[7] Takaya Y., Noda T., Nakajima I. (2014). Electrocardiographic predictors of response to cardiac resynchronization therapy in patients with intraventricular conduction delay. Circ J.

[8] Ueda N., Noda T., Nakajima I. (2020). Clinical impact of left ventricular paced conduction disturbance in cardiac resynchronization therapy. Heart Rhythm.

[9] Varma N., Stadler R.W., Ghosh S., Kloppe A. (2017). Influence of automatic frequent pace-timing adjustments on effective left ventricular pacing during cardiac resynchronization therapy. EP Europace.

[10] Matia R., Hernandez-Madrid A., Klepfer R.N., Ghosh S., Sanchez-Huete G., Moreno J. (2017). A new electrogram-based diagnostic algorithm to improve the left ventricular effective pacing detection corrected a non-response to cardiac resynchronization therapy pacing. EP Europace.

[11] Hernandez-Madrid A., Facchin D., Klepfer R.N. (2017). Device pacing diagnostics overestimate effective cardiac resynchronization therapy pacing results of the hOLter for Efficacy analysis of CRT (OLE CRT) study. Heart Rhythm.

[12] Thibault B., Ducharme A., Harel F. (2011). Left ventricular versus simultaneous biventricular pacing in patients with heart failure and a QRS complex ≥120 milliseconds. Circulation.

[13] Trucco E., Tolosana J.M., Arbelo E. (2018). Improvement of reverse remodeling using electrocardiogram fusion-optimized intervals in cardiac resynchronization therapy: a randomized study. JACC Clin Electrophysiol.

[14] Pujol-Lopez M., San Antonio R., Mont L. (2019). Electrocardiographic optimization techniques in resynchronization therapy. EP Europace.

[15] Oka S., Ueda N., Ishibashi K. (2023). Significance of effective cardiac resynchronization therapy pacing for clinical responses: an analysis based on the effective cardiac resynchronization therapy algorithm. Heart Rhythm.

[16] Alasti M., Rangasamy K., Healy S., Adam D., Kotschet E. (2017). Loss of cardiac resynchronization therapy in a patient with a biventricular implantable cardioverter-defibrillator. J Arrhythm.

